# Expression of 4-Hydroxynonenal (4-HNE) and Heme Oxygenase-1 (HO-1) in the Kidneys of *Plasmodium berghei*-Infected Mice

**DOI:** 10.1155/2020/8813654

**Published:** 2020-10-23

**Authors:** Prasit Na-Ek, Chuchard Punsawad

**Affiliations:** School of Medicine, Walailak University, Tha Sala District, Nakhon Si Thammarat 80160, Thailand

## Abstract

Acute kidney injury (AKI) is one of the most serious complications of severe *Plasmodium falciparum* malaria, but the exact pathogenic mechanisms of AKI in *P. falciparum* infection have not been clearly elucidated. We hypothesized that oxidative stress is a potential mediator of acute tubular necrosis in *P. falciparum*-infected kidneys. Therefore, this study aimed to investigate the histopathological changes and markers of oxidative stress in kidney tissues from mice with experimental malaria. DBA/2 mice were divided into two groups: the mice in the malaria*-*infected group (*n* = 10) were intraperitoneally injected with 1 × 10^6^*P. berghei* ANKA-infected red blood cells, and the mice in the control group (*n* = 10) were intraperitoneally injected with a single dose of 0.85% normal saline. Kidney sections were collected and used for histopathological examination and the investigation of 4-hydroxynonenal (4-HNE) and heme oxygenase-1 (HO-1) expression through immunohistochemistry staining. The histopathology study revealed that the *P. berghei*-infected kidneys exhibited a greater area of tubular necrosis than those of the control group (*p* < 0.05). The positive staining scores for 4-HNE and HO-1 expression in tubular epithelial cells of the *P. berghei*-infected group were significantly higher than those found for the control group (*p* < 0.05). In addition, significant positive correlations were found between the tubular necrosis score and the positive staining scores for 4-HNE and HO-1 in the kidneys from the *P. berghei*-infected group. In conclusion, this finding demonstrates that increased expression of 4-HNE and HO-1 might be involved in the pathogenesis of acute tubular damage in the kidneys during malaria infection. Our results provide new insights into the pathogenesis of malaria-associated AKI and might provide guidelines for the future development of a therapeutic intervention for malaria.

## 1. Introduction

Malaria remains a life-threatening disease throughout the world, particularly in tropical regions, and resulted in 405,000 deaths in 2019 [1]. *Plasmodium falciparum* is the main cause of severe malaria, which causes dysfunction in one or several crucial organs [2]. Acute kidney injury (AKI), which is one of the most hypercritical complications experienced by patients with severe malaria, affects 40% of adult patients and is associated with a high mortality rate [3]. At present, the pathogenesis and precise mechanism of *P. falciparum-*induced AKI remain unclear although previous studies have hypothesized involving an excessive immune response, mechanical obstruction by infected red blood cells, fluid loss due to multiple mechanisms, and alterations in the renal microcirculation [4–6]. The pathophysiology of malaria involves oxidative stress arising from many possible sources, such as red blood cells infected with *P. falciparum* [7], human phagocytic cells activated by *P. falciparum* [8], elevation of the oxidative host enzyme levels [9], and the occurrence of ischemia and reperfusion syndrome during malarial paroxysm and cytoadherence [10]. Additional possible sources could be reactive oxygen species (ROS) production by parasites and the action of prooxidants and antimalarial drugs [10]. The plasma levels of toxic heme and oxidized low-density lipoprotein (LDL), which are products generated by oxidative stress during the development of malaria-associated AKI, are during *P. berghei* ANKA malarial infection [11]. Several oxidative stress products, such 4-hydroxynonenal (4-HNE), heme oxygenase-1 (HO-1), and oxidized-LDL, are also found in the blood circulation and many organs after malaria infection [11, 12]. However, the presence of oxidative stress in kidney tissue has not been widely determined.

In general, we hypothesized that oxidative stress is correlated with the kidney pathology of *P. berghei-*infected mice. Therefore, this study aimed to investigate the histopathologic changes and the expression of 4-HNE and HO-1 as markers of oxidative stress in *P. berghei-*infected mice. In addition, the correlations between histopathological changes and the expression of 4-HNE and HO-1 in kidney tissues of *P. berghei-*infected mice were analyzed.

## 2. Materials and Methods

### 2.1. Animals and Malaria Infection

Male DBA/2 mice (aged 6–8 weeks) were purchased from BioLASCO Co., Ltd. (Taipei, Taiwan). The mice were housed in two standard polycarbonate cages with wood-shaving bedding (five mice per cage) and were given ad libitum access to feed and unrestricted access to water. The animals were maintained under controlled temperature and humidity conditions and exposed to a 12 h light/12 h dark cycle. The mice in the infected group (*n* = 10) were intraperitoneally infected with 1 × 10^6^*P. berghei* ANKA-infected red blood cells, as previously described [13]. The malaria parasites were obtained through BEI Resources, NIAID, NIH: *P. berghei*, strain ANKA, MRA-311, contributed by Thomas F. McCutchan. The control mice (*n* = 10) received a single dose of 0.85% normal saline. Starting after 3 days, the presence and degree of parasitemia were determined daily by Giemsa staining and expressed as the percentage of infected red blood cells. According to the evidence on the DBA/2 mice that died with hyperparasitemia on day 12 after infection, this study was designed to collect the kidney sample at day 13 [14]. The mice were euthanized, and the kidneys were collected to investigate the histopathological changes through hematoxylin and eosin (H&E) staining and to determine the expression of 4-HNE and HO-1 through immunohistochemistry staining. The study protocol was reviewed and approved by the Animal Ethics Committee at Walailak University (protocol no. 019/2018).

### 2.2. Histopathological Analysis of the Kidneys

All tissues were prepared as described in a previous study [15]. In brief, the tissues were fixed in 10% buffered formalin for at least 24–48 h and then subjected to standard histological processing for paraffin-embedded sections. The kidney tissues were sectioned to a thickness of 4 *μ*m and stained with hematoxylin and eosin (H&E). A histopathological scoring method was used to quantify the changes in the kidney tissues under a light microscope. The degree of histopathological alterations was scored based on the following variables as described in a previous report [16]: evidence of parasitized red blood cells (PRBCs) within the capillaries, congestion, tubular necrosis, and leukocyte infiltration. In brief, each histologic parameter was evaluated by examining all microscopic fields of each slide, and a five-point scale was used as follows: 0, <10%; 1, 10–25%; 2, 26–50%; 3, 51–75%; and 4, >75% of the section affected. Tubular necrosis was evaluated by determining the percentage of tubules exhibiting epithelial necrosis, loss of the brush border, cast formation, and tubular dilation. At least ten microscopic fields (200x) for each mouse in both groups were separately examined by two observers blinded to the experimental group allocation. To quantify the overall histopathological changes in the kidney tissues, all the scores were added to obtain a combined score in the range of 0 to 16: a score of 0 indicated no injury, whereas a score of 16 indicated maximal severity.

### 2.3. Immunohistochemical Staining for 4-HNE and HO-1

The expression of 4-HNE and HO-1 was assessed by immunohistochemistry staining as described previously [15]. In brief, the kidney sections were deparaffinized in xylene and rehydrated through a descending gradient of ethanol. Endogenous peroxidase activities were inhibited by incubation in 3% hydrogen peroxide. To enhance the reactivity of 4-HNE and HO-1 antibodies in formalin-fixed tissues, the kidney sections were subjected to heat-mediated antigen retrieval with 0.1 M Tris-HCl buffer, pH 9.0, in a microwave oven for 10 min, and nonspecific binding was blocked by incubation with normal goat serum for 30 min. The tissues were then incubated with one of the following primary antibodies overnight at 4°C: rabbit polyclonal anti-4-HNE (1 : 100 dilution) (Cat no. ab46545, Abcam, Cambridge, UK) or rabbit polyclonal anti-HO-1 (1 : 100 dilution) (Cat no. ab13243, Abcam, Cambridge, UK). After three washes with Tris-buffered saline (TBS), the sections were incubated with biotinylated goat anti-rabbit IgG (Vector Laboratories, Inc., Burlingame, CA, USA) for 30 min. The bound rabbit antibody was detected by incubation with an avidin biotinylated horseradish peroxidase complex (Vector Laboratories, Inc., Burlingame, CA) for 30 min, and the activity of peroxidase (brown color) was then observed using 3,3'‐diaminobenzidine (DAB) as the chromogen (Vector Laboratories, CA, USA). The target for 4-HNE and HO-1 expression was localized in the cytoplasmic compartment of the cells. Subsequently, the stained sections were counterstained with hematoxylin and observed under a light microscope.

### 2.4. Evaluation of Immunohistochemical Staining

The protein expression of 4-HNE and HO-1 was assessed using a grading system based on tubular epithelial cells with positive staining of the cytoplasm. 4-HNE- and HO-1-positive tubules were scored semiquantitatively by estimating the percentage of tubular epithelial cells expressing 4-HNE and HO-1 per field, and these scores were defined as follows based on the staining criteria according to a previous study with minor modification [17]: 0, nonstained epithelial tubular cells or up to <10% tubular epithelial cells with positive staining; 1, 10.1% to 25% tubular epithelial cells with positive staining; 2, 25.1% to 50% tubular epithelial cells with positive staining; 3, 50.1% to 75% tubular epithelial cells with positive staining; and 4, 75.1% to 100% tubular epithelial cells with positive staining. All the slides were examined by two independent observers using a double-blind review method.

### 2.5. Statistical Analysis

All the data are expressed as the means ± standard deviation (SD) unless otherwise indicated. The normality of all the data was tested using the Shapiro–Wilks test. Differences between groups were compared using Student's *t*-test. Differences were considered statistically significant if *p* < 0.05. The correlations among the data were analyzed by Pearson's correlation, and differences were considered significant if *p* < 0.05.

## 3. Results

### 3.1. Parasitemia Development

**Figure 1:**
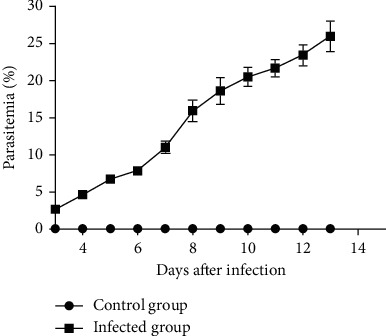
Parasitemia in *P. berghei*-infected (*n* = 10) and the control groups (*n* = 10).

Mice infected with *P. berghei* developed 2.65 ± 0.34 parasitemia at day 3 after infection. The parasitemia rose rapidly reaching the mean peak of 25.94 ± 4.46 at day 13 (Figure 1). However, no infected mice died spontaneously, but all of them were sacrificed on the 13th day after infection.

### 3.2. Histopathological Changes in the Kidneys

**Figure 2:**
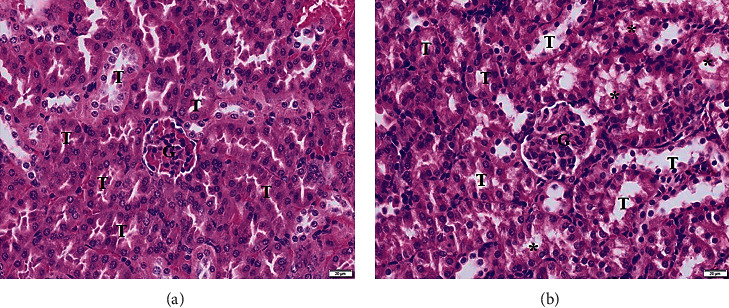
Hematoxylin and eosin (H&E)-stained kidney sections from the control and *P. berghei*-infected groups. The kidney section of the control group shows a normal glomerulus and typical tubular cells (a). The kidney section of the *P. berghei*-infected group presented an area of tubular necrosis (asterisks) and a dilated lumen of renal tubules (b). 200x magniﬁcation, bar = 20 *μ*m. G, glomerulus; T, renal tubule.

As demonstrated by H&E staining, the kidney sections obtained from the control group showed normal histology features with glomeruli (G) and renal tubules (T) (Figure 2(a)). In contrast, the kidney sections of the *P. berghei*-infected group revealed marked disruption of the normal kidney arrangement with predominant necrosis of tubular epithelial cells (Figure 2(b)). Vasodilation of the glomerulus and mild infiltration of inflammatory cells were also observed in the kidney sections of the *P. berghei*-infected group (Figure 2(b)). As demonstrated through a semiquantitative analysis, the degree of tubular necrosis in the *P. berghei*-infected group (3.20 ± 0.40) was significantly higher than that in the control group (0.00 ± 0.00) (*p* < 0.05), and the degree of leukocyte infiltration in the *P. berghei*-infected group (1.30 ± 0.10) was significantly higher than that in the control group (0.00 ± 0.00) (*p* < 0.05). In addition, the mean score of the overall histopathological changes in the kidney tissues was significantly higher in the *P. berghei*-infected group (4.46 ± 0.42) than in the control group (0.00 ± 0.00).

### 3.3. Immunohistochemical Staining for 4-HNE and HO-1

**Figure 3:**
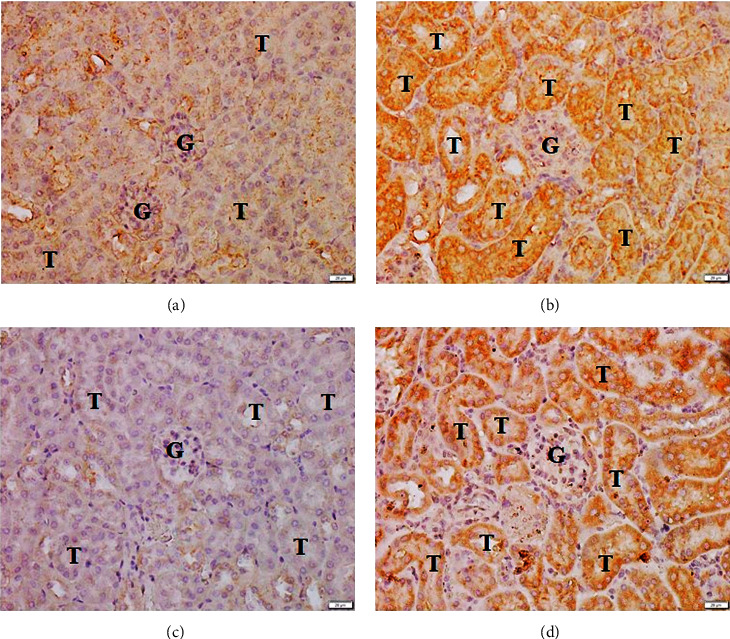
Immunohistochemistry staining for 4-HNE and HO-1 in kidney sections from the control and *P. berghei*-infected groups. The kidney sections of the control group showed very weak staining for 4-HNE (a) and HO-1 (c). The kidney sections of *P. berghei*-infected mice showed strong positive staining for 4-HNE (b) and HO-1 (d). 200x magnification, bar = 20 *μ*m. G, glomerulus; T, renal tubule.

Immunohistochemistry staining for 4-HNE and HO-1 was performed to investigate the oxidative stress markers in the kidney sections. Positive staining of 4-HNE and HO-1 proteins was localized in the cytoplasm. In addition, positive staining for 4-HNE was mostly observed in the tubular epithelial cells of the *P. berghei*-infected group (Figure 3(b)), whereas few positive cells were detected among the tubular epithelial cells of the control group (Figure 3(a)). Positive staining for HO-1 was found in the tubular epithelial cells of the *P. berghei*-infected group (Figure 3(b)). In addition, the glomeruli of both *P. berghei*-infected mice and control mice showed negative staining for 4-HNE and HO-1 proteins (Figure 3).

**Figure 4:**
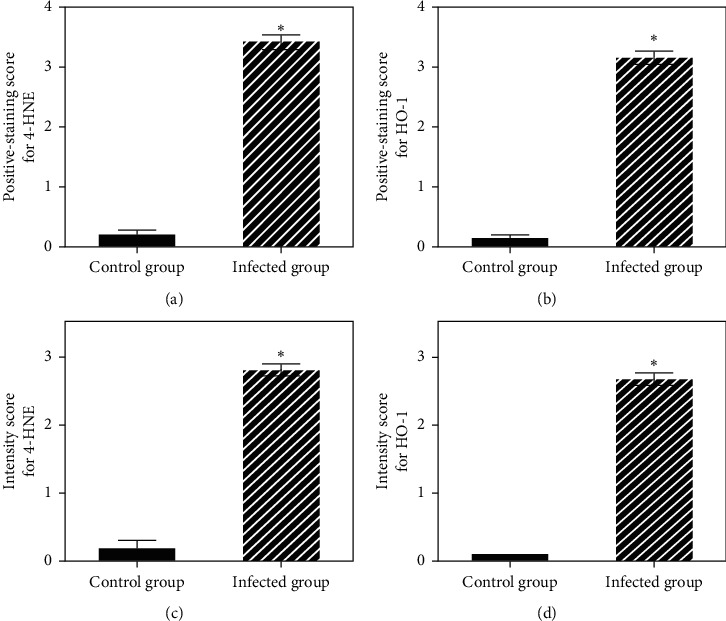
Semiquantitative analysis of 4-HNE and HO-1 expression in kidney sections from the control (a) and *P. berghei*-infected groups (b). ^*∗*^Significant (*p* < 0.05) difference compared with the control group.

A semiquantitative analysis revealed that the positive staining scores for 4-HNE and HO-1 in the kidneys of the *P. berghei*-infected group were significantly higher than those obtained for the control kidneys group (*p* < 0.05) (Figures 4(a) and 4(b)). Interestingly, the 4-HNE and HO-1 staining intensities were also significantly higher in the kidneys of the *P. berghei*-infected group than in those of the control group (*p* < 0.05) (Figures 4(c) and 4(d)).

### 3.4. Correlation between Histopathological Changes and 4-HNE and HO-1 Expression

**Figure 5:**
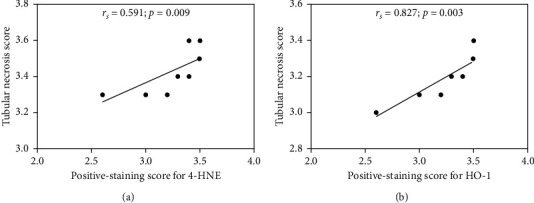
Correlation between the tubular necrosis score and the positive staining scores for 4-HNE (a) and HO-1 (b) in the kidney sections of the *P. berghei*-infected group (*n* = 10). The correlations among the data were analyzed by Pearson's correlation, and significance was indicated by *p* < 0.05.

According to a previous report investigated in the kidney tissues from P. falciparum malaria patients demonstrated that tubular necrosis was found to be highest in *P*. falciparum malaria patients with AKI and significantly correlated with the presence of apoptosis [15]. Based on our histopathological analysis, tubular necrosis was major histopathological change observed in the kidney of the *P. berghei*-infected group. Therefore, we next investigated whether expression of 4-HNE and HO-1 correlated with tubular necrosis. Using Pearson's correlation, significant positive correlations were found between the tubular necrosis score and the positive staining scores for 4-HNE (Pearson's correlation *r*_*s*_ = 0.591, *p*=0.009) and HO-1 (Pearson's correlation *r*_s_ = 0.827, *p*=0.003) in the kidneys from the *P. berghei*-infected group (Figures 5(a) and 5(b)).

## 4. Discussion

The present study provides evidence describing the histopathological changes that occur in the kidney of an experimental murine model of severe malaria, and this analysis revealed the presence of tubular necrosis and infiltration of inflammatory cells consistent with malaria-associated AKI in *P. falciparum* malaria. The impairment of renal function during malaria infection has been noted in previous clinical reports [18–20], and this impairment is an important life-threatening complication of malaria infection that goes beyond the classical clinical symptoms of plasmodium.

Oxidative stress is considered a determinant of disease severity during *P. falciparum* malaria infection and increases during disease progression [21–23]. Malaria-associated AKI has been proposed as a consequence of parasite adhesion and an exacerbated immune response against the products of oxidative stress released during *Plasmodium* infection [24]. The destruction of red blood cells during the blood stage of infection increases the high levels of toxic-free heme in the blood circulation, which can induce oxidative stress through the production of hydroxyl radicals via the Fenton/Haber–Weiss reaction [25]. A previous study reported that increases in ROS increase the function of human dendritic cells (DCs) that support the host inflammatory response, which is correlated with an increase in the inflammatory response and the subsequent destruction of tubular cells [26].

The immunohistochemical analysis performed in this study revealed that the positive staining scores for 4-HNE and HO-1 in the renal tubular epithelial cells of the *P. berghei*-infected group were significantly higher than those found for the control group. Previous studies have shown that the level of 4-HNE in the blood circulation is increased during plasmodium infection and that 4-HNE is involved in the progression to severe malaria with immunosuppression and anemia [27]. 4-HNE is the final product of oxidative stress and lipoperoxidation [28] generated by hemozoin-containing parasitized red blood cells and hemozoin-fed phagocytes [29]. 4-HNE binds to extracellular domains of red blood cell membrane proteins and might diffuse to neighboring cells to form stable 4-HNE-protein conjugates [30]. This study reveals that the levels of 4-HNE-conjugates are increased in tubular epithelial cells of *P. berghei*-infected mice, which suggests that 4-HNE might play a role in malaria-associated kidney injury. Regarding HO-1, the recent data revealed that HO-1 expression was induced during AKI. HO-1 provides cytoprotective effects to the kidney via modulating the immune response, regulating cell cycle, and catabolizing heme, a potent prooxidant, and thus, HO-1 may serve as a therapeutic target in AKI [31]. Our study showed that AKI in *P. berghei*-infected mice was also associated with increased HO-1 expression. Under strong oxidative stress, HO-1, which is an enzyme that catabolizes heme into labile ferrous iron, carbon monoxide (CO), and biliverdin (BV), is rapidly upregulated to handle the increased levels of free heme [32]. The increased level of HO-1 in the kidneys of *P. berghei*-infected mice in this study might also be mediated by the increase in free heme. In addition, a high level of HO-1 was also found in patients with severe malaria and in brain tissues from patients with cerebral malaria induced by prostaglandin (PG) D2 [33].

## 5. Conclusions

This study demonstrates that the expression levels of 4-HNE and HO-1 are increased in the kidneys of *P. berghei-*infected mice and that these increases are positively related to the tubular necrosis score. Our results suggest that high 4-HNE and HO-1 expressions might be involved in the pathogenesis of acute tubular damage in the kidney during malaria infection.

## Data Availability

The data used to support the findings of this study are included within the article.
